# Familial risk of sinus node dysfunction indicating pacemaker implantation: a nationwide cohort study

**DOI:** 10.1093/europace/euae287

**Published:** 2024-11-13

**Authors:** Morten Krogh Christiansen, Erik Thorlund Parner, Jens Brock Johansen, Jens Cosedis Nielsen, Henrik Kjærulf Jensen

**Affiliations:** Department of Cardiology, Viborg Regional Hospital, Heibergs alle 2K, DK-8800 Viborg, Denmark; Department of Clinical Medicine, Health, Aarhus University, Palle Juul-Jensens Boulevard 99, DK-8200 Aarhus N, Denmark; Department of Public Health, Section of Biostatistics, Aarhus University, Aarhus, Denmark; Department of Cardiology, Odense University Hospital, Odense, Denmark; Department of Clinical Medicine, Health, Aarhus University, Palle Juul-Jensens Boulevard 99, DK-8200 Aarhus N, Denmark; Department of Cardiology, Aarhus University Hospital, Aarhus, Denmark; Department of Clinical Medicine, Health, Aarhus University, Palle Juul-Jensens Boulevard 99, DK-8200 Aarhus N, Denmark; Department of Cardiology, Aarhus University Hospital, Aarhus, Denmark

**Keywords:** Family history, Genetics, Epidemiology, Sinus node dysfunction, Pacemaker, Mortality

## Abstract

**Aims:**

We aimed to investigate the risk of sinus node dysfunction (SND) indicating cardiac pacing and mortality in first-degree relatives to patients with a pacemaker implanted on this indication and assess the effect of onset-age on disease risk.

**Methods and results:**

In this nationwide register-based study, we used the Danish Civil Registration Registry to establish family structures and merged data with the Danish National Patient Registry and the Danish Pacemaker and ICD Registry containing information on all pacemakers implanted due to SND in Denmark. We followed 6 027 090 individuals born after 1954 in the period between 1982 and 2022 (180 775 041 person-years) among whom 2.477 pacemakers were implanted due to SND. The adjusted rate ratio (RR) of pacemaker-treated SND was 2.9 (2.4–3.6) for individuals having any father, mother, or sibling with a pacemaker implanted on this indication compared with the general population (derived cumulative incidence at the age of 68 years: 0.79 and 0.27%, respectively). This risk was inversely proportional to implantation age in the index person [≤60 years: RR = 5.5 (3.4–9.0)]. Overall, mortality was similar between individuals having a father, mother, or sibling with SND and the general population, but higher for relatives to index persons with an early onset [≤60 years: RR = 1.22 (1.05–1.41)].

**Conclusion:**

First-degree relatives to SND patients are at increased risk of SND with risk being inversely associated with pacemaker implantation age in the index person. Mortality in first-degree relatives was comparable with the general population, although subgroup findings suggest an increased mortality among individuals with a family history of early-onset SND.

## Graphical Abstract

**Graphical Abstract:**
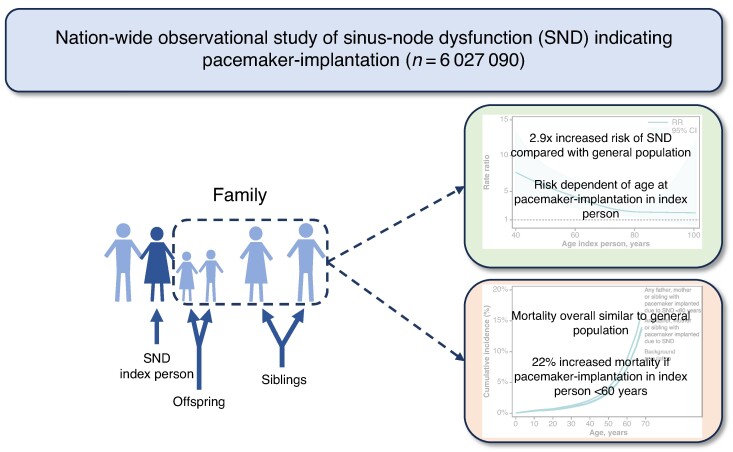

**What’s new?**

- Individuals with a family history of sinus node dysfunction (SND) had a 2.9-fold increased risk of pacemaker implantation on this indication, with risk inversely associated to the age of pacemaker implantation in the index person.
- Overall, mortality was comparable to the general population, although significantly increased in individuals with a family history of early-onset SND.

## Introduction

Sinus node dysfunction (SND) is a heterogeneous clinical syndrome characterized by inappropriate sinus bradycardia, sinoatrial block, and/or chronotropic incompetence. Although rates of pacemaker implantation due to SND differ substantially across countries, the disorder comprises one of the most common indications for pacemaker implantation worldwide.^1,2^ The disease most often presents in the elderly population and is associated with underlying structural heart disease, which may cause SND through development of ischaemia, fibrosis, or remodelling of the atrial wall.^3,4^ However, rare forms of familial SND have been reported, in which pathogenic genetic variants, encoding cardiac ion channels such as *SCN5A*^5,6^ and *HCN4*,^7,8^ have been identified through candidate gene and linkage analysis methods. Sinus node dysfunction in such families may also be seen as part of an arrhythmic syndrome, possibly affecting the prognosis among family members.^6,9^ More recently, genome-wide association studies have identified a number of common genetic variants implicated in SND suggesting various other ion channels and cytoskeletal proteins to be implicated in disease development.^10,11^ Although these variants each confer a relatively small increase in SND risk, they are frequently found in the population,^10^ potentially indicating that SND may have a common inheritance. To date, there have been no population-based studies estimating the familial risk of SND. Therefore, we aimed to investigate the risk of SND indicating cardiac pacing and mortality in first-degree relatives to patients (index persons) with a pacemaker implanted on this indication and to assess the effect of age at implantation in the index person on disease risk.

## Methods

### Design, sinus node dysfunction definition, and study population

This was a nationwide retrospective register-based cohort study. We included individuals born in 1954 or thereafter. Sinus node dysfunction was defined to be present from the time a pacemaker was implanted on this indication. We then compared the rates of pacemaker implantation due to SND in first-degree relatives of pacemaker-treated SND patients with the rates in the general population in Denmark between 1 January 1982 and 31 August 2022.

### Data sources

At the time of birth or immigration, all individuals living in Denmark are given a unique and permanent civil registration number. This number is used in national registries, which allows cross-linking of information between registries. We used data from three registries: the Danish Civil Registration System, the Danish Pacemaker and ICD Registry, and the Danish National Patient Registry.

The Danish Civil Registration System contains information on date of birth, sex, maternal and paternal identity, and vital status on all people living in Denmark.^12^ Among persons born in Denmark in 1954 or later, the information on maternal and paternal identity is almost complete.^12,13^ We used these parental links to construct familial relations.

The Danish Pacemaker and ICD Registry is a national clinical database that contains prospective data from all pacemaker implantations performed in Denmark from 1 January 1982 and onwards.^14^ Upon pacemaker implantation, clinical and hardware data are entered into the database by the implanting physician. This information includes indication for pacemaker implantation, which for SND is further specified as SND with/without pause and bradycardia-tachycardia syndrome. A total of 49 417 brady-pacemaker implantations were registered by 31 August 2022, of which the indication was specified in 42 309 cases. In Denmark, indications for pacemaker implantation follow contemporary guidelines from the European Society of Cardiology.^1^ The extracted data sample for this study contained all pacemaker implantations due to SND from the beginning of the registry up to 31 August 2022.

The Danish National Patient Registry contains data entered by the treating physician on all patients discharged from non-psychiatric hospitals since 1977 and on emergency department and outpatient clinic contacts since 1995.^15^ Upon discharge or contact to the outpatient clinic, one primary diagnosis and optional secondary diagnoses and procedure codes are registered in the database. We used diagnosis and procedure codes to identify selected comorbidities that may aggregate in families and be of possible importance for the development of SND (see Supplementary material online, *Table S1*).

### Statistical analysis

We used data from the Civil Registration Registry and the Danish Pacemaker and ICD Registry to identify (i) offspring of mothers with a pacemaker implanted due to SND, (ii) offspring of fathers with a pacemaker implanted due to SND, and (iii) siblings to a person with a pacemaker implanted due to SND. Siblings were considered to be in the same sibship if they had the same mother. Subpopulations of relatives to index persons were constructed as follows: for offspring of a mother or father (the index person) with SND, their risk time in the subpopulation started at the time of the pacemaker implantation in the parent. For siblings in a sibship, the first sibling to experience a pacemaker implantation due to SND was defined as the index person and the remaining siblings’ risk time in the subpopulation started at the time of the pacemaker implantation in the index person. For the general population as well as each of the subpopulations, we then calculated the cumulative incidence of pacemaker implantation due to SND with age as the underlying timescale. We further calculated the expected number of pacemaker implantations based on the rates in the general population which were used as a reference in the Poisson regression to calculate the rate ratios (RRs). The corresponding 95% confidence intervals (CIs) were calculated based on robust standard errors taking into account the non-independent observations due to familial clustering. All RRs were modelled as a function of sex, calendar time (year 1982–1989, 1990–1994, …, 2010–2014, and 2015+), and age (0–44, 45–49, 50–54, …, 85–89, and 90+ years). In multivariable analyses, we further adjusted for selected comorbidities (see Supplementary material online, *Table S1*), modelled as time-dependent variables. The cumulative incidence was estimated based on the Poisson model using the method of Benichou and Gail.^16^ Pacemaker implantation due to SND ≤ 60 years of age was chosen as a definition of early-onset SND for which subgroup sizes were judged to be reasonable. To illustrate the effect of SND onset age in the index person, we modelled the RRs as a function of the age at pacemaker implantation using restricted cubic splines with four knots.

This study was reported to the repository of the Central Denmark Region (record number 1-16-02-64-22). In Denmark, no ethical approval nor informed consent from the study participants is needed for registry-based studies. All data were analysed on the Danish Health Data Authority server using STATA (version 18.0) with access only to pseudonymized data. In agreement with the terms of use, we do not report on groups of less than five individuals.

## Results

**Figure 1:**
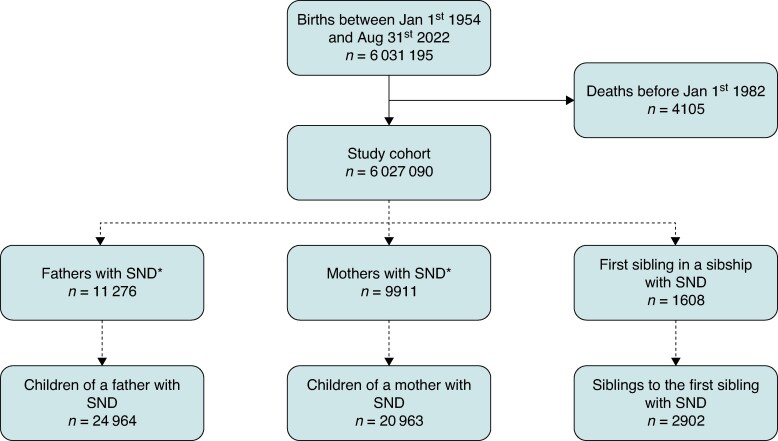
Cohort illustration. *All parents with a pacemaker implanted due to SND were considered index persons if their children are among the study cohort. Parents born before 1954, however, are not part of the study cohort themselves and are thus not included in the populations that form the basis for calculating the rate ratios. SND, sinus node dysfunction.

We included 6 027 090 individuals born in 1954 or thereafter (*Figure 1*) in the study cohort.

Parents with a pacemaker implanted due to SND were registered as index persons disregarding their year of birth if their children were among the study cohort, although parents born before 1954 were not part of the study cohort themselves. The study cohort was followed for 180 775 041 person-years between 1 January 1982 and 31 August 2022 among whom 2.477 pacemakers were implanted due to SND in the time period. Baseline characteristics of index persons and relatives are displayed in *Tables 1* and *2*. The median age at pacemaker implantation was 75, 72, and 50 years among index mothers, fathers, and siblings, respectively. Cardiovascular comorbidities were prevalent among these patients, in particular, hypertension, atrial fibrillation or flutter, and coronary artery disease (*Table 1*).

**Table 1:** Baseline characteristics of index persons with SND at the time of pacemaker implantation

	Index father	Index mother	Index sibling
Total	11 276 (100.0)	9911 (100.0)	1608 (100.0)
Age, years			
≤30	67 (0.6)	68 (0.7)	224 (13.9)
30–40	129 (1.1)	74 (0.7)	185 (11.5)
40–50	375 (3.3)	213 (2.1)	381 (23.7)
50–60	1167 (10.3)	662 (6.7)	550 (34.2)
60–70	2791 (24.8)	1951 (19.7)	268 (16.7)
70–80	4370 (38.8)	3992 (40.3)	*n* < 5
>80	2377 (21.1)	2951 (29.8)	*n* < 5
Male sex			1033 (64.2)
Comorbidities			
Hypertension	4483 (39.8)	4829 (48.7)	300 (18.7)
Diabetes mellitus	1432 (12.7)	1205 (12.2)	94 (5.8)
Heart failure	1767 (15.7)	1362 (13.7)	148 (9.2)
Coronary artery disease	4268 (37.9)	2995 (30.2)	202 (12.6)
Atrial fibrillation or flutter	5148 (45.7)	5246 (52.9)	402 (25.0)
Valvular heart disease	1289 (11.4)	1282 (12.9)	114 (7.1)
Stroke	1268 (11.2)	1084 (10.9)	64 (4.0)
Cardiac surgery	3621 (32.1)	1998 (20.2)	313 (19.5)

Data are presented as *n* (%). All parents with SND were considered index persons if their children are among the study cohort. Parents born before 1954, however, are not part of the study cohort themselves and are thus not included in the populations that form the basis for calculating the rate ratios.

SND, sinus node dysfunction.

**Table 2:** Baseline characteristics of relatives to index persons with SND at the time of pacemaker implantation in the index person

	Offspring to index father	Offspring to index mother	Sibling to index sibling
Total	24 777^a^ (100.0)	20 836^a^ (100.0)	2 893^a^ (100.0)
Age, years			
≤30	4466 (18.0)	1743 (8.4)	384 (13.3)
30–40	6308 (25.5)	3409 (16.4)	383 (13.2)
40–50	8655 (34.9)	7536 (36.2)	808 (27.9)
50–60	4897 (19.8)	7040 (33.8)	1003 (34.7)
60–68	451 (1.8)	1108 (5.3)	315 (10.9)
Male sex	12 499 (50.4)	10 675 (51.2)	1494 (51.6)
Comorbidities			
Hypertension	690 (2.8)	1029 (4.9)	202 (7.0)
Diabetes mellitus	368 (1.5)	447 (2.1)	79 (2.7)
Heart failure	73 (0.3)	130 (0.6)	39 (1.3)
Coronary artery disease	370 (1.5)	549 (2.6)	108 (3.7)
Atrial fibrillation or flutter	177 (0.7)	259 (1.2)	53 (1.8)
Valvular heart disease	93 (0.4)	106 (0.5)	30 (1.0)
Stroke	158 (0.6)	218 (1.0)	43 (1.5)
Cardiac surgery	229 (0.9)	274 (1.3)	45 (1.6)

Data are presented as *n* (%).

SND, sinus node dysfunction.

^a^Only relatives, who were born and alive at the time of pacemaker implantation in the index person, are included in the table.

### Risk of sinus node dysfunction indicating pacemaker implantation

**Figure 2:**
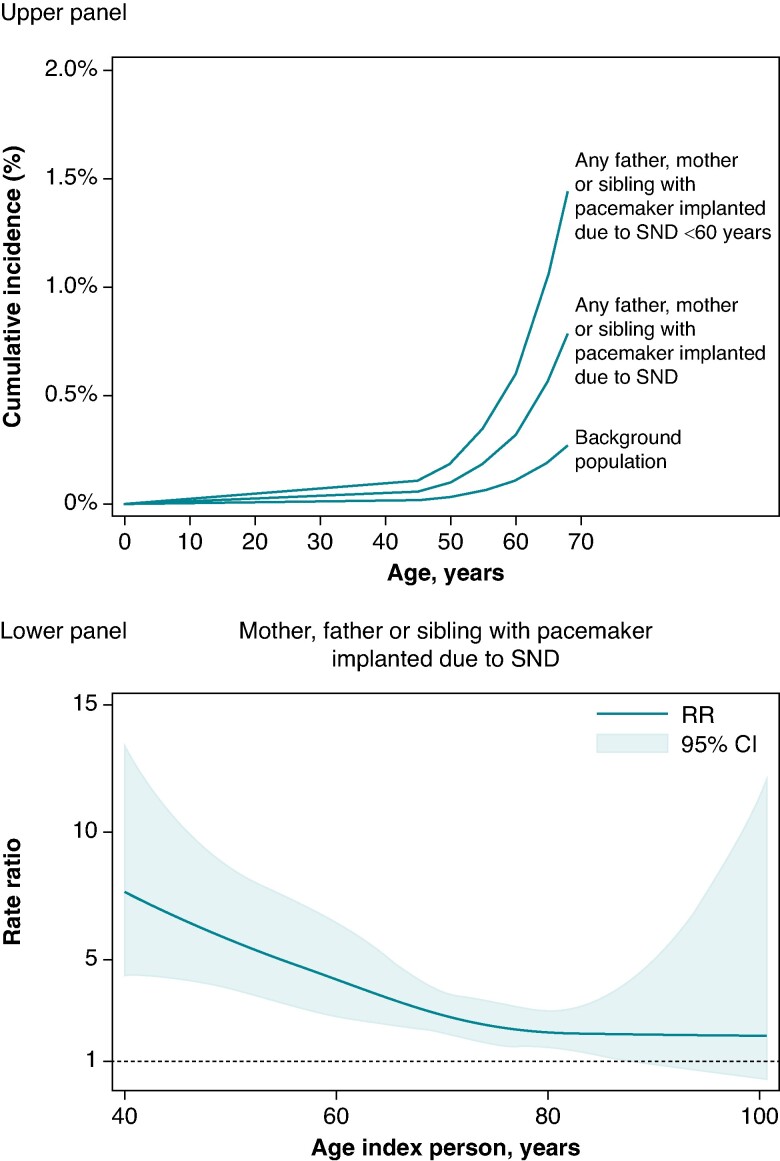
Risk of SND indicating cardiac pacing according to age at pacemaker implantation in index person. Upper panel: the cumulative incidence of SND indicating pacemaker implantation in relatives to an index person with a pacemaker implanted on this indication. The cumulative incidence was calculated based on the rates in the general population and the adjusted rate ratios. Lower panel: spline model displaying the rate ratio of SND indicating cardiac pacing in relatives to index persons compared with the general population as a function of age at pacemaker implantation in the index person. The rate ratio was adjusted for hypertension, diabetes mellitus, heart failure, coronary artery disease, atrial fibrillation or flutter, valvular heart disease, and stroke. SND, sinus node dysfunction.

The cumulative incidence of pacemaker implantation due to SND was 0.03% (95% CI 0.03–0.04%) and 0.27% (95% CI 0.25–0.29%) at 50 and 68 years in the general population (*Figure 2*). Among individuals having a father, mother, or sibling with a pacemaker implanted due to SND, the cumulative incidence was 0.10% (95% CI 0.06–0.14%) and 0.79% (95% CI 0.49–1.09%) at 50 and 68 years corresponding to an adjusted 2.9-fold (95% CI 2.4–3.6) increased risk (*Table 3*). This risk was increased across the entire age spectrum of age at pacemaker implantation in the index person, but significantly higher if pacemaker implantation in the index person occurred at an early age. Accordingly, among individuals having a father, mother, or sibling with a pacemaker implanted ≤60 years of age, the cumulative incidence of pacemaker implantation due to SND was 0.19% (95% CI 0.11–0.27%) and 1.45% (95% CI 0.84–2.06%) at 50 and 68 years corresponding to an adjusted 5.5-fold (95% CI 3.4–9.0) increased risk compared with the general population. Subgroup analyses revealed no substantial differences in risk depending on whether the index person was a mother, father, or sibling, when the age at pacemaker implantation in the index person was accounted for (*Table 3*; Supplementary material online, *Figure S1*). Similarly, the increased risk of pacemaker implantation due to SND was comparable for male and female relatives to an index person (see Supplementary material online, *Tables S2* and *S3*). An increased risk of pacemaker implantation due to SND was observed in relatives regardless of the type of SND in the index person (see Supplementary material online, *Tables S4* and *S5*). Having any father, mother, or sibling with the bradycardia-tachycardia form of SND was associated with a 2.1-fold (95% CI 1.3–3.5) increased risk of pacemaker implantation, whereas relatives to index patients with other forms of SND had a 3.2-fold (95% CI 2.5–4.1) increased risk compared with the general population.

**Table 3:** Risk of SND indicating cardiac pacing in first-degree relatives to an index person with a pacemaker implanted on this indication

		Model 1		Model 2	
	No. of events	Rate ratio (95% CI)	*P*-value	Rate ratio (95% CI)	*P*-value
Any father, mother, or sibling	89	3.4 (2.7–4.2)	<0.001	2.9 (2.4–3.6)	<0.001
Any father, mother, or sibling > 60 years	69	2.9 (2.3–3.7)	<0.001	2.6 (2.0–3.3)	<0.001
Any father, mother, or sibling ≤ 60 years	20	7.3 (4.5–11.8)	<0.001	5.5 (3.4–9.0)	<0.001
Father	36	2.8 (2.0–4.0)	<0.001	2.5 (1.8–3.6)	<0.001
Father > 60 years	29	2.5 (1.7–3.6)	<0.001	2.2 (1.6–3.2)	<0.001
Father ≤ 60 years	7	7.0 (3.0–16.3)	<0.001	5.7 (2.5–13.4)	<0.001
Mother	45	3.4 (2.5–4.6)	<0.001	3.0 (2.2–4.0)	<0.001
Mother > 60 years	43	3.4 (2.5–4.6)	<0.001	3.0 (2.2–4.1)	<0.001
Mother ≤ 60 years	*n* < 5	NA	NA	NA	NA
Siblings	13	8.8 (4.9–15.8)	<0.001	6.4 (3.5–11.7)	<0.001

In Model 1, rate ratios were modelled as a function of age, sex, and calendar time. In Model 2, we further adjusted for hypertension, diabetes mellitus, heart failure, coronary artery disease, atrial fibrillation or flutter, valvular heart disease, stroke, and cardiac surgery modelled as time-dependent variables.

SND, sinus node dysfunction.

### Mortality

**Figure 3:**
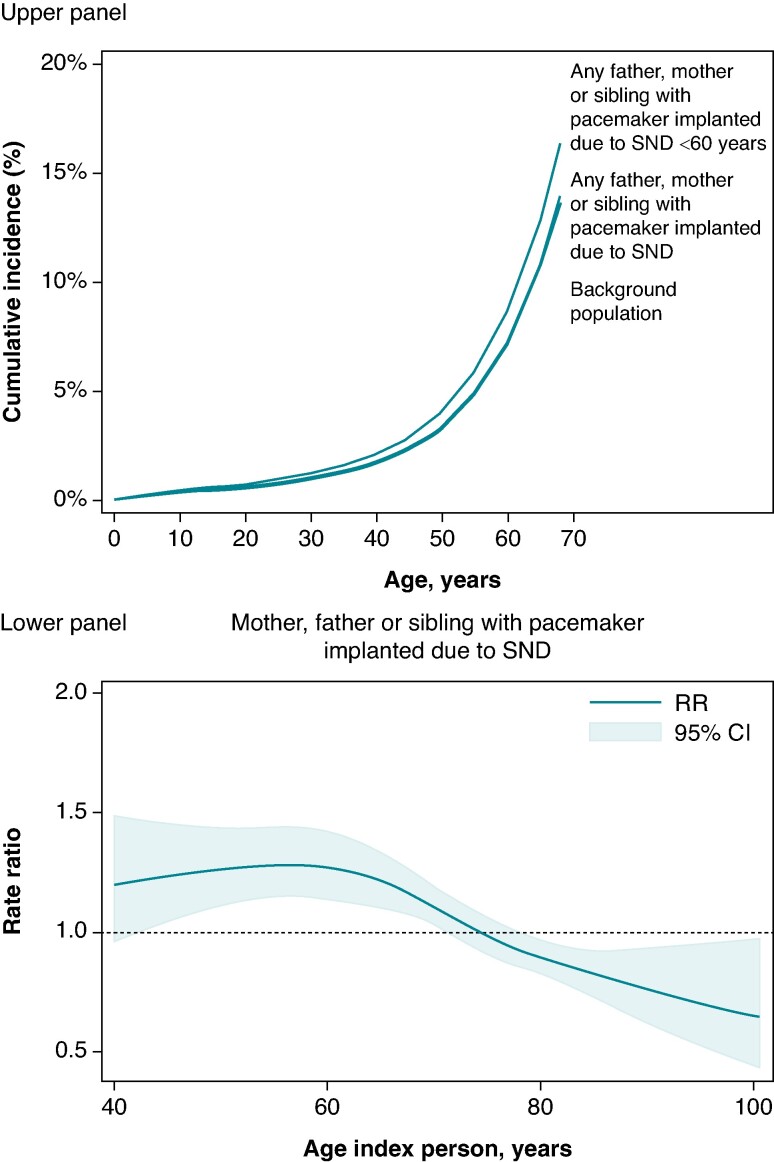
Mortality in relatives according to age at pacemaker implantation due to SND in index person. Upper panel: the cumulative mortality in relatives to an index person with a pacemaker implanted due to SND. The cumulative mortality was calculated based on the rates in the general population and the adjusted rate ratios. Lower panel: spline model displaying the mortality rate ratio in relatives to index persons with SND compared with the general population as a function of age at pacemaker implantation in the index person. The rate ratio was adjusted for hypertension, diabetes mellitus, heart failure, coronary artery disease, atrial fibrillation or flutter, valvular heart disease, and stroke. SND, sinus node dysfunction.

The cumulative mortality was 3.3% (95% CI 3.3–3.3%) increasing to 13.6% (95% CI 13.5–13.7%) at 50 and 68 years in the general population, which was almost identical to that observed among individuals having any father, mother, or sibling with a pacemaker implanted due to SND [adjusted RR 1.02 (95% CI 0.97–1.08)] (*Table 4*). However, the spline model illustrated an inverse proportional relationship between age at pacemaker implantation in the index person and mortality among relatives. Thus, mortality at 50 and 68 years was 4.0% (95% CI 2.1–5.9%) and 16.3% (95% CI 9.1–23.6%) among individuals having a father, mother or sibling with a pacemaker implanted due to SND ≤ 60 years of age corresponding to an adjusted 1.22-fold (95% CI 1.05–1.41) increased mortality (*Figure 3*). To explore the cause of the increased mortality in patients with a family history of early-onset SND, we performed an additional analysis investigating the risk of a combined endpoint of ventricular tachyarrhythmia or cardiac arrest based on diagnosis codes from the Danish National Patient Registry (see Supplementary material online, *Table S1*). Having a father, mother, or sibling with a pacemaker implanted due to SND was associated with an adjusted 1.2-fold (95% CI 1.0–1.3) increased risk of the combined endpoint (see Supplementary material online, *Table S6*), which was driven by an increased risk among individuals with a family history of pacemaker implantation ≤ 60 years of age [adjusted RR 1.5 (1.1–2.0)].

**Table 4:** Risk of all-cause mortality in first-degree relatives to an index person with a pacemaker implanted due to SND

		Model 1		Model 2	
	No. of events	Rate ratio (95% CI)	*P*-value	Rate ratio (95% CI)	*P*-value
Any father, mother, or sibling	1373	1.06 (1.01–1.12)	0.03	1.02 (0.97–1.08)	0.45
Any father, mother, or sibling >60 years	1178	1.03 (0.97–1.09)	0.33	0.99 (0.94–1.05)	0.86
Any father, mother, or sibling ≤ 60 years	195	1.31 (1.13–1.52)	<0.001	1.22 (1.05–1.41)	0.009
Father	631	1.00 (0.92–1.08)	0.93	0.97 (0.89–1.05)	0.45
Father > 60 years	554	0.97 (0.89–1.05)	0.43	0.94 (0.87–1.03)	0.17
Father ≤ 60 years	77	1.29 (1.02–1.63)	0.03	1.22 (0.97–1.54)	0.10
Mother	704	1.12 (1.04–1.21)	0.004	1.07 (0.99–1.15)	0.10
Mother > 60 years	653	1.09 (1.01–1.18)	0.03	1.04 (0.96–1.13)	0.29
Mother ≤ 60 years	51	1.55 (1.17–2.06)	0.002	1.47 (1.12–1.95)	0.006
Siblings	84	1.20 (0.96–1.49)	0.11	1.10 (0.88–1.37)	0.42

In Model 1, rate ratios were modelled as a function of age, sex, and calendar time. In Model 2, we further adjusted for hypertension, diabetes mellitus, heart failure, coronary artery disease, atrial fibrillation or flutter, valvular heart disease, stroke, and cardiac surgery modelled as time-dependent variables.

SND, sinus node dysfunction.

## Discussion

In the present study, we investigated familial risk of developing SND indicating pacemaker implantation. Overall, we found a 2.9-fold increased risk of pacemaker implantation due to SND if a mother, father, or sibling had a pacemaker implanted on this indication. The risk was increased regardless of sex, family relationship, and type of SND, but increased significantly the younger the index person was at the time of implantation suggesting a generally substantial genetic contribution to SND development, a contribution which is particularly strong in families where pacemakers are implanted on this indication at an early age. Of interest, the risk estimate remained largely unchanged after multivariable adjustment for potential cardiovascular mediators suggesting a substantial direct genetic basis of SND.

Recently, scientific interest exploring the yield of genetic testing in young patients with pacemaker-indicating cardiac conduction defects (including both atrioventricular block and SND) has emerged.^17–20^ However, knowledge on risk among family members to SND patients remains scarce. One study has investigated familial risk for cardiac conduction defects.^21^ They found a 1.7-fold increased risk of developing cardiac conduction defects among first-degree relatives of an affected person. However, the study was limited by the fact that the endpoint was based on International Classification of Diseases 10th Revision codes with a significant under-reporting of SND, which only accounted for 10% of the total number of pacemaker implantations, and the fact that familial risk of SND was not separately investigated. Although familial forms of SND have been recognized for many years,^22^ our study is, to our knowledge, the first to investigate the familial risk of developing SND and the associated mortality.

In general, there is no evidence that bradycardia due to SND is associated with a poor outcome.^1,23,24^ Therefore, the increased mortality among relatives of early-onset SND patients may seem surprising. Sinus node dysfunction is a feature seen in several inherited arrhythmic syndromes such as catecholaminergic polymorphic ventricular tachycardia,^25^ and in particular *SCN5A* gene-associated diseases such as long QT syndrome^26,27^ and Brugada syndrome.^9,28^ These rare inherited syndromes, often underdiagnosed,^29^ are associated with an increased risk of early sudden death, and it is possible that they contribute to explain the increased mortality in families with early-onset SND. This hypothesis may be supported by the observation of an increased risk of ventricular tachyarrhythmias or cardiac arrest in these families. However, this is likely not the whole explanation, since the observed difference in mortality between the general population and relatives of patients with a pacemaker implanted due to SND ≤ 60 years of age was 2.7% at the age of 68 years whereas the corresponding difference in pacemaker implantations was only 1.2%. Another contributing factor may be that SND patients more commonly suffer from comorbidities and have an increased mortality compared with the general population.^30,31^ Accordingly, we observed a high burden of cardiovascular comorbidity among these patients. Although we adjusted for cardiovascular diseases of potential interest, it is possible that other diseases aggregate particularly in families with early-onset SND and that these diseases contribute to explain the increased mortality. In contrast to an increased mortality among relatives of early-onset SND patients, there was a trend towards a lower mortality among relatives of patients with pacemakers implanted due to SND later in life. The reason for this finding is probably that individuals who survive to have a pacemaker implanted at an older age exhibit longevity to which their relatives are thus predisposed.

Our findings may have a number of implications. From a medical knowledge perspective, they provide increased insight into SND, emphasizing that SND is a hereditary disease with familial risk estimates that are on a par with other common cardiac diseases such as coronary artery disease^13,32^ and atrial fibrillation.^33,34^ A heredity that applies to the bradycardia-tachycardia syndrome as well as other forms of SND. From a clinical perspective, our findings may contribute to raise the suspicion of SND as a cause of cardiac symptoms in patients with a family history of SND. In particular, this applies to families with early-onset SND or families in which several individuals are affected by SND. In these cases, our findings highlight the possibility of an underlying genetic cause among whom genetic and familial evaluation may be warranted.

### Strengths and limitations

A major strength of our study is the use of nationwide registries with almost complete data on parental links and comorbidities. Validation studies have reported that the positive predictive values of the cardiovascular diagnoses used in our analyses are high.^35,36^ An additional strength is that our SND definition required that a pacemaker was implanted on this indication in order for SND to be clinically significant. However, it might be possible that using a wider definition of SND could have produced other risk estimates.

A number of limitations should be mentioned. The parental links were not established in the Danish Civil Registration System until the 1950s. Given the available data, relatives to index persons with SND had a maximum age of 68 years at the end of the study period. Since the majority of pacemaker implantations due to SND occur at an older age, the majority of implantations in the cohort were performed in later years. Nevertheless, the population was still relatively young to develop a SND indication for pacemaker implantation which means that the effect of a family history on developing SND at older age cannot be determined. In addition, we were only able to identify pairs of siblings who were born after 1954, whereas there were no such ‘restrictions’ on parents of individuals in the study population. This means that the index sibling age was restricted to a maximum of 68 years, whereas there was no upper age limit for an index parent, resulting in an average lower age for index siblings.

Another limitation is that there may be mediators or confounders that we did not adjust for in the regression analyses, and thus, residual confounding cannot be excluded. Data in the Danish Patient Registry does not include diagnoses made by general practitioners. Since an isolated diagnosis of hypertension or diabetes often does not lead to a referral to a hospital, these diagnoses are most likely under-reported in the dataset. Sinus node dysfunction diagnoses and pacemaker indications were all verified by cardiac electrophysiologists, and therefore, the risk of misclassification is likely modest. However, in such cases, this would be assumed to happen in random which would push the estimates on familial risk towards the null. Finally, we did not have access to the diagnoses on the death certificates, and therefore, we cannot further qualify the cause of the increased mortality observed among relatives to patients with early-onset SND.

## Conclusion

First-degree relatives to patients with a pacemaker implanted due to SND carried a 2.9-fold increased risk of pacemaker implantation on the same indication. The risk was inversely associated with age of pacemaker implantation in the index person but independent of sex, family relationship, and type of SND. Overall, mortality in first-degree relatives was comparable with the general population; however, subgroup analyses indicated a higher mortality as well as an increased risk of ventricular tachyarrhythmias and cardiac arrest for relatives to index persons with an early onset. This latter observation, including the underlying cause, needs further exploration.

## Supplementary Material

euae287_Supplementary_Data

## Data Availability

The data that support this study will be shared upon reasonable request to the corresponding author.
