# Supplementary material for: Familial risk of sinus node dysfunction indicating pacemaker implantation: a nationwide cohort study
Source: Europace. 2024 Nov 13;26(12):euae287. doi: 10.1093/europace/euae287 (PMC11630507; doi:10.1093/europace/euae287)
Supplement: euae287_Supplementary_Data [file euae287_supplementary_data.docx]

# Supplemental Material

## Supplemental Table S1: Definitions of diseases based on diagnosis- and procedure codes in the Danish National Patient Registry

|  | **Diagnosis codes** | |
| --- | --- | --- |
|  | **ICD-8** | **ICD-10** |
| Hypertension | 400-404 | I10-I15, I67.4 |
| Diabetes mellitus | 249, 250 | E10, E11, H36.0 |
| Heart failure | 42709, 42710, 42711, 42719, 42899,78249 | I50, I11.0, I13.0, I13.2, I42.0, I42.6, I42.7, I42.8, I42.9 |
| Coronary artery disease | 410-414 | I20-I21, I23-I25 |
| Atrial fibrillation or flutter | 42793, 42794 | I48 |
| Valvular heart disease | 394-398 | I05, I06, I07, I08.0, I09.8, I34-I37, I51.1A, Q22 |
| Stroke | 431, 433-434 | I61, I63-I64 |
| Ventricular tachyarrhythmia or cardiac arrest | 42797, 42791, 42727, 42797 | I47.0, I47.2, I49.0, I46 |
|  | **Procedure codes** | |
|  | **DCSP** | **NOMESCO** |
| Cardiac surgery | opr30000 – opr32990 | KF |

DCSP = Danish Classification of Surgical Procedures; ICD = International Classification of Diseases; NOMESCO = Nordic Medico-Statistical Committee Classification of Surgical Procedures

## Supplemental Table S2: Risk of pacemaker-indicating SND in male relatives to an index person with a pacemaker implanted on this indication

|  |  | **Model 1** |  | **Model 2** |  |
| --- | --- | --- | --- | --- | --- |
|  | **No events** | **Rate Ratio (95% CI)** | **P-value** | **Rate Ratio (95% CI)** | **P-value** |
| Any father, mother or sibling | 56 | 3.2 (2.5-4.2) | <0.001 | 2.8 (2.1-3.6) | <0.001 |
| Any father, mother or sibling >60 years | 42 | 2.7 (2.0-3.7) | <0.001 | 2.4 (1.8-3.2) | <0.001 |
| Any father, mother or sibling ≤60 years | 14 | 7.8 (4.4-13.6) | <0.001 | 5.5 (3.1-9.8) | <0.001 |
| Father | 25 | 3.0 (2.0-4.6) | <0.001 | 2.7 (1.8-4.0) | <0.001 |
| Father >60 years | 19 | 2.5 (1.6-3.9) | <0.001 | 2.2 (1.4-3.5) | <0.001 |
| Father ≤60 years | 6 | 9.2 (3.7-23.1) | <0.001 | 7.1 (2.8-18.0) | <0.001 |
| Mother | 25 | 2.9 (1.9-4.2) | <0.001 | 2.5 (1.7-3.7) | <0.001 |
| Mother >60 years | 25 | 3.0 (2.0-4.4) | <0.001 | 2.6 (1.8-3.9) | <0.001 |
| Mother ≤60 years | n<5 | NA | NA | NA | NA |
| Siblings | 9 | 9.2 (4.8-17.6) | <0.001 | 6.4 (3.2-12.5) | <0.001 |

In Model 1 rate ratios were modeled as a function of age, sex, and calendar time. In Model 2 we further adjusted for hypertension, diabetes mellitus, heart failure, coronary artery disease, atrial fibrillation or flutter, valvular heart disease, stroke, and cardiac surgery modeled as time-dependent variables. SND = sinus node dysfunction.

## Supplemental Table S3: Risk of SND in female relatives to an index person with a pacemaker implanted on this indication

|  |  | **Model 1** |  | **Model 2** |  |
| --- | --- | --- | --- | --- | --- |
|  | **No events** | **Rate Ratio (95% CI)** | **P-value** | **Rate Ratio (95% CI)** | **P-value** |
| Any father, mother or sibling | 33 | 3.6 (2.6-5.1) | <0.001 | 3.2 (2.3-4.5) | <0.001 |
| Any father, mother or sibling >60 years | 27 | 3.3 (2.3-4.8) | <0.001 | 3.0 (2.0-4.3) | <0.001 |
| Any father, mother or sibling ≤60 years | 6 | 6.4 (2.9-14.4) | <0.001 | 5.4 (2.4-12.2) | <0.001 |
| Father | 11 | 2.5 (1.4-4.5) | 0.003 | 2.3 (1.3-4.2) | 0.005 |
| Father >60 years | 10 | 2.4 (1.3-4.5) | 0.005 | 2.3 (1.2-4.3) | 0.009 |
| Father ≤60 years | n<5 | NA | NA | NA | NA |
| Mother | 20 | 4.5 (2.9-6.9) | <0.001 | 3.9 (2.5-6.0) | <0.001 |
| Mother >60 years | 18 | 4.2 (2.7-6.7) | <0.001 | 3.6 (2.3-5.8) | <0.001 |
| Mother ≤60 years | n<5 | NA | NA | NA | NA |
| Siblings | n<5 | NA | NA | NA | NA |

In Model 1 rate ratios were modeled as a function of age, sex, and calendar time. In Model 2 we further adjusted for hypertension, diabetes mellitus, heart failure, coronary artery disease, atrial fibrillation or flutter, valvular heart disease, stroke, and cardiac surgery modeled as time-dependent variables. SND = sinus node dysfunction.

## Supplemental Table S4: Risk of bradycardia-tachycardia form of SND in relatives to an index person

|  |  | **Model 1** |  | **Model 2** |  |
| --- | --- | --- | --- | --- | --- |
|  | **No events** | **Rate Ratio (95% CI)** | **P-value** | **Rate Ratio (95% CI)** | **P-value** |
| Any father, mother or sibling | 15 | 2.8 (1.7-4.6) | <0.001 | 2.1 (1.3-3.5) | 0.004 |
| Any father, mother or sibling >60 years | 14 | 2.8 (1.7-4.8) | <0.001 | 2.2 (1.3-3.8) | 0.003 |
| Any father, mother or sibling ≤60 years | n<5 | NA | NA | NA | NA |
| Father | 5 | 2.0 (0.8-4.8) | 0.12 | 1.6 (0.7-3.8) | 0.31 |
| Father >60 years | 5 | 2.1 (0.9-5.1) | 0.09 | 1.7 (0.7-4.1) | 0.24 |
| Father ≤60 years | n<5 | NA | NA | NA | NA |
| Mother | 9 | 3.2 (1.7-6.2) | <0.001 | 2.4 (1.3-4.7) | 0.007 |
| Mother >60 years | 9 | 3.3 (1.7-6.4) | <0.001 | 2.5 (1.3-4.9) | 0.005 |
| Mother ≤60 years | n<5 | NA | NA | NA | NA |
| Siblings | n<5 | NA | NA | NA | NA |

In Model 1 rate ratios were modeled as a function of age, sex, and calendar time. In Model 2 we further adjusted for hypertension, diabetes mellitus, heart failure, coronary artery disease, atrial fibrillation or flutter, valvular heart disease, stroke, and cardiac surgery modeled as time-dependent variables. SND = sinus node dysfunction.

## Supplemental Table S5: Risk of non-bradycardia-tachycardia forms of SND in relatives to an index person

|  |  | **Model 1** |  | **Model 2** |  |
| --- | --- | --- | --- | --- | --- |
|  | **No events** | **Rate Ratio (95% CI)** | **P-value** | **Rate Ratio (95% CI)** | **P-value** |
| Any father, mother or sibling | 74 | 3.5 (2.8-4.4) | <0.001 | 3.2 (2.5-4.1) | <0.001 |
| Any father, mother or sibling >60 years | 55 | 2.9 (2.2-3.8) | <0.001 | 2.7 (2.1-3.6) | <0.001 |
| Any father, mother or sibling ≤60 years | 19 | 8.3 (5.1-13.7) | <0.001 | 6.8 (4.2-11.2) | <0.001 |
| Father | 32 | 3.1 (2.2-4.5) | <0.001 | 2.9 (2.0-4.2) | <0.001 |
| Father >60 years | 25 | 2.7 (1.8-3.9) | <0.001 | 2.5 (1.7-3.7) | <0.001 |
| Father ≤60 years | 7 | 8.2 (3.6-19.0) | <0.001 | 7.0 (3.0-16.2) | <0.001 |
| Mother | 36 | 3.4 (2.5-4.8) | <0.001 | 3.1 (2.3-4.4) | <0.001 |
| Mother >60 years | 34 | 3.4 (2.4-4.8) | <0.001 | 3.1 (2.2-4.4) | <0.001 |
| Mother ≤60 years | n<5 | NA | NA | NA | NA |
| Siblings | 12 | 10.2 (5.5-18.8) | <0.001 | 8.2 (4.4-15.3) | <0.001 |

In Model 1 rate ratios were modeled as a function of age, sex, and calendar time. In Model 2 we further adjusted for hypertension, diabetes mellitus, heart failure, coronary artery disease, atrial fibrillation or flutter, valvular heart disease, stroke, and cardiac surgery modeled as time-dependent variables. SND = sinus node dysfunction.

## Supplemental Table S6: Risk of ventricular tachyarrhythmia or cardiac arrest in relatives to index persons with a pacemaker implanted due to SND

|  |  | **Model 1** |  | **Model 2** |  |
| --- | --- | --- | --- | --- | --- |
|  | **No events** | **Rate Ratio (95% CI)** | **P-value** | **Rate Ratio (95% CI)** | **P-value** |
| Any father, mother or sibling | 247 | 1.2 (1.1-1.4) | 0.001 | 1.2 (1.0-1.3) | 0.02 |
| Any father, mother or sibling >60 years | 202 | 1.1 (1.0-1.3) | 0.06 | 1.1 (1.0-1.3) | 0.15 |
| Any father, mother or sibling ≤60 years | 45 | 1.8 (1.3-2.4) | <0.001 | 1.5 (1.1-2.0) | 0.009 |
| Father | 122 | 1.2 (1.0-1.5) | 0.03 | 1.2 (1.0-1.4) | 0.06 |
| Father >60 years | 107 | 1.2 (1.0-1.4) | 0.07 | 1.2 (1.0-1.4) | 0.10 |
| Father ≤60 years | 15 | 1.5 (0.9-2.4) | 0.14 | 1.3 (0.8-2.2) | 0.29 |
| Mother | 119 | 1.2 (1.0-1.5) | 0.02 | 1.2 (1.0-1.4) | 0.12 |
| Mother >60 years | 99 | 1.1 (0.9-1.3) | 0.39 | 1.0 (0.8-1.3) | 0.77 |
| Mother ≤60 years | 20 | 3.5 (2.2-5.4) | <0.001 | 3.0 (1.9-4.8) | <0.001 |
| Siblings | 10 | 0.9 (0.5-1.7) | 0.78 | 0.7 (0.4-1.3) | 0.29 |

In Model 1 rate ratios were modeled as a function of age, sex, and calendar time. In Model 2 we further adjusted for hypertension, diabetes mellitus, heart failure, coronary artery disease, atrial fibrillation or flutter, valvular heart disease, stroke, and cardiac surgery modeled as time-dependent variables. SND = sinus node dysfunction.

## Supplemental Figure 1: Risk of SND according to age at SND onset in index person


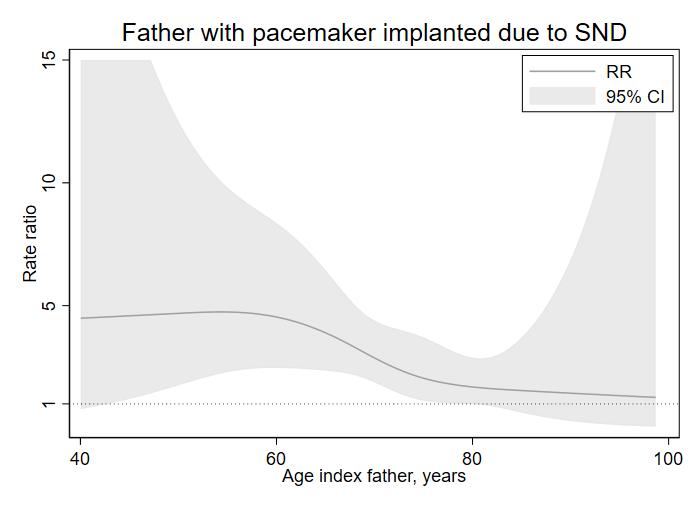


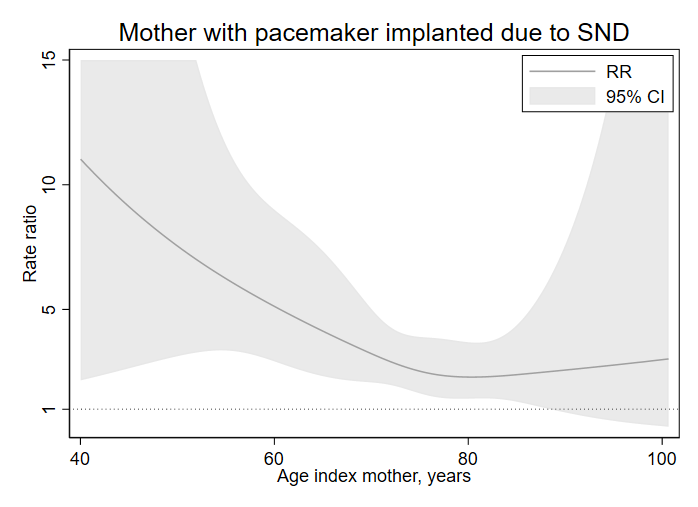


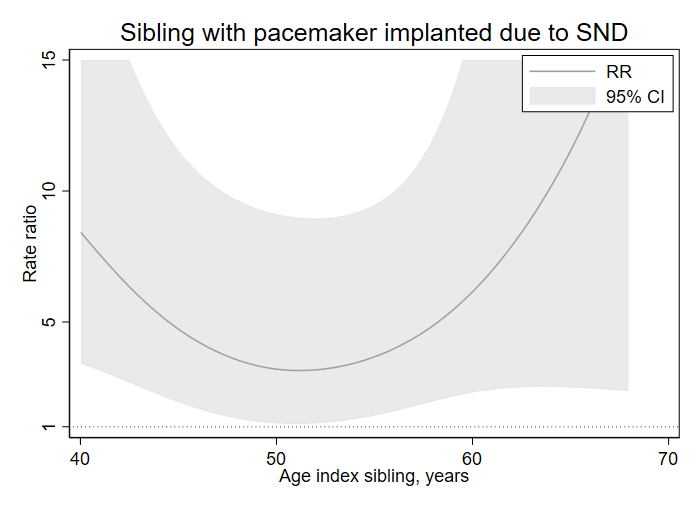


SND = sinus node dysfunction.
